# Interhemispheric functional connectivity: an fMRI study in callosotomized patients

**DOI:** 10.3389/fnhum.2024.1363098

**Published:** 2024-05-15

**Authors:** Ilaria Marcantoni, Giusi Piccolantonio, Mojgan Ghoushi, Marco Valenti, Luca Reversi, Francesco Mariotti, Nicoletta Foschi, Simona Lattanzi, Laura Burattini, Mara Fabri, Gabriele Polonara

**Affiliations:** ^1^Dipartimento di Ingegneria dell'Informazione, Università Politecnica delle Marche, Ancona, Italy; ^2^Dipartimento di Ingegneria per Medicina di Innovazione, Università di Verona, Verona, Italy; ^3^Dipartimento di Scienze Radiologiche, Azienda Ospedaliera-Universitaria Umberto I, Ancona, Italy; ^4^Dipartimento di Scienze Neurologiche, Azienda Ospedaliera-Universitaria Umberto I, Ancona, Italy; ^5^Dipartimento di Medicina Sperimentale e Clinica, Università Politecnica delle Marche, Ancona, Italy; ^6^Dipartimento di Scienze della Vita e dell’Ambiente, Università Politecnica delle Marche, Ancona, Italy; ^7^Dipartimento di Scienze Cliniche Specialistiche e Odontostomatologiche, Università Politecnica delle Marche, Ancona, Italy

**Keywords:** callosotomy, interhemispheric connectivity, lateralization, resting-state, functional connectivity

## Abstract

**Introduction:**

Functional connectivity (FC) is defined in terms of temporal correlations between physiological signals, which mainly depend upon structural (axonal) connectivity; it is commonly studied using functional magnetic resonance imaging (fMRI). Interhemispheric FC appears mostly supported by the corpus callosum (CC), although several studies investigating this aspect have not provided conclusive evidence. In this context, patients in whom the CC was resected for therapeutic reasons (split-brain patients) provide a unique opportunity for research into this issue. The present study was aimed at investigating with resting-state fMRI the interhemispheric FC in six epileptic patients who have undergone surgical resection of the CC.

**Methods:**

The analysis was performed using fMRI of the Brain Software Library; the evaluation of interhemispheric FC and the recognition of the resting-state networks (RSNs) were performed using probabilistic independent component analysis.

**Results:**

Generally, bilateral brain activation was often observed in primary sensory RSNs, while in the associative areas, such as those composing the default mode and fronto-parietal networks, the activation was often unilateral.

**Discussion:**

These results suggest that even in the absence of the CC, some interhemispheric communication is still present. This residual FC might be supported through extra-callosal pathways that are likely subcortical, making it possible for some interhemispheric integration. Further studies are needed to confirm these conclusions.

## Introduction

In recent years, neuroscience research has seen growing interest in the study of the brain resting-state activity, usually studied through functional magnetic resonance imaging (fMRI). The resting-state brain activity is defined as the intrinsic activity of the brain, which occurs spontaneously, i.e., in the absence of purposely given sensitive and/or cognitive external stimuli (Smitha et al., 2017). This intrinsic activity reveals itself in the fMRI data through the correlation of blood oxygenated level-dependent (BOLD) time series, defining the so-called resting-state networks (RSNs) (Uddin, 2013). RSNs are groups of functionally coherent but anatomically separated brain regions. The most common RSNs were 8, first described by Beckmann and coworkers (Beckmann et al., 2005): medial visual area (MVA), lateral visual area (LVA), auditory cortices (AUD), sensory-motor area (SMA), visuo-spatial system or default mode network (DMN), executive control (EXE), frontoparietal area left (FPA-l), and frontoparietal area right (FPA-r). Later, Smith and coworkers identified two more networks by analyzing fMRI data from 36 healthy subjects during resting-state (Smith et al., 2009): occipital visual area (OVA) and cerebellum (CER). From a physiological point of view, during rest, the left and right hemispheric regions of different functional networks do not remain quiescent but prove a high level of spontaneous neural activation with a high correlation among several non-adjacent brain regions (van den Heuvel and Hulshoff Pol, 2010). Such activity can be used to characterize network dynamics without inducing a specific cognitive task to determine brain activity (Uddin, 2013). Because of this, the brain seems to be mainly managed by its intrinsic activity, while sensitive or cognitive external stimuli simply have the role of modulating it.

The interhemispheric connectivity can be functional (FC) or structural (SC). FC is supposed to be supported by SC, i.e., the white matter (WM) axonal bundles of the brain. The knowledge of the correspondence between SC and FC would allow to better understand the physiological processes of the brain. The most evident characteristic of resting-state FC is the symmetry of correlation respect to the midsagittal plane, which tends to be particularly high between corresponding foci in the two hemispheres; in this case, the FC is said homotopic (Salvador et al., 2005; Stark et al., 2008). The SC is supported mainly by the corpus callosum (CC), which is the major commissure physically linking the two hemispheres, and the anterior (AC), posterior (PC), and hippocampal commissures.

The AC is the most ventral of the three and primarily connects olfactory and amygdaloid regions. Despite its small size relative to the human CC, the AC is the most evolutionarily ancient of these routes, as it is present in all vertebrates thus far examined, connecting brain areas essential for survival (Fenlon et al., 2021). Studies in eutherians, including humans, have further demonstrated that the areas containing neurons projecting through the AC encompass the anterior olfactory nucleus through its anterior branch, the piriform and entorhinal cortices, and the lateral/temporal portions of the neocortex through its posterior branch and amygdaloid areas such as the nucleus of the lateral olfactory tract and cortical amygdala through the stria terminalis branch (Fenlon et al., 2021), possibly connecting slightly different circuits (e.g., homotopic, heterotopic neocortical, and heterotopic hippocampal, respectively).

The hippocampal commissure, as its name implies, connects hippocampal and associated areas (e.g., entorhinal cortex) and lies immediately ventral to the CC. This commissure is the main efferent pathway of the hippocampus, connecting the hippocampal formation to structures beyond the temporal lobe, which is crucial in declarative memory formation and consolidation (Fenlon et al., 2021).

The PC is one of the diencephalic commissures, whose functions are probably related to involuntary eye movements (Fenlon et al., 2021); PC connects the superior colliculi, which are involved in the bilateral pupillary light reflex (Ozdemir, 2015). In typical development, the PC is an exclusively subcortical, mesodiencephalic bundle that makes direct connections with the nucleus of Darkschewitsch and the red nucleus, as well as with the habenular nuclei (Keene, 1938; Tovar-Moll et al., 2014).

The thalamic commissure (TC) has been recently reported in many mammalian species (rodents, New and Old World primates; Szczupak et al., 2023) as an additional interhemispheric axonal fiber pathway that connects the cortex to the contralateral thalamus. The TC develops during the embryonic period, forming anatomical and functionally active connections of the cortex with the contralateral thalamus. The presence of TC in humans has been identified up to now in subjects with brain malformations. These results pose the TC as an important fiber pathway in the primate brain, allowing for more robust interhemispheric connectivity and synchrony and serving as an alternative commissural route in developmental brain malformations.

The CC is the main interhemispheric commissure, arising in the brain of placental mammals (Aboitiz and Montiel, 2003) as an elongated midline structure composed of 200–800 million horizontally running fibers interconnecting homotopical and heterotopical cortical areas (Innocenti, 1986; Innocenti et al., 2022). The function of the CC has been investigated since the 16th century, and the first description of bundles of axons passing through the callosal WM and connecting the two hemispheres (Manzoni, 2011) dates back to the 18th century. The known CC functions include the interhemispheric exchange of information, integration of inputs reaching one or both hemispheres, facilitation of some cortical functions, and inhibition of others (Wahl et al., 2007; Koch et al., 2011; Innocenti et al., 2022). The size of the human CC positively correlates with intelligence (Einstein’s CC was thicker than normal; Men et al., 2014) and its integrity is essential for cognitive performances; therefore, CC resection and microstructural or developmental alterations are often associated with cognitive decline (O’Sullivan et al., 2001; Schulte et al., 2005; Paul, 2011; Passamonti et al., 2014; Westerhausen and Karud, 2018).

The earliest hypotheses on the function of the human CC came from studies of split-brain patients, subjects whose CC was partially or completely resected to prevent the diffusion of epileptic seizures (Gazzaniga, 2000, 2005). Patients with total or partial resection involving the posterior CC suffered from disconnection syndrome (Geschwind, 1965a,b; Berlucchi, 2014), whereas in those with partial anterior resection, the disconnection could be evidenced only by specific tests (Gordon et al., 1971; Berlucchi, 2012). The emerging idea is that the CC connects the cerebral hemispheres and provides interhemispheric integration and information transfer. In addition, from these studies, the idea of a functional topographical organization of the CC, corresponding to the morphological subdivisions described above, started to appear.

Consequently, the CC is assumed to be the most responsible for the symmetry of FC, although this commissure has been shown to contain many fibers connecting non-homotopic regions (Innocenti et al., 2022; Szczupak et al., 2023). Nevertheless, data demonstrating the actual role of CC in interhemispheric connectivity are still insufficient, and those present in the literature are not conclusive (Johnston et al., 2008; Uddin et al., 2008; Pizoli et al., 2011; Roland et al., 2017). Patients who underwent callosotomy, i.e., in whom the CC was resected for therapeutic reasons (split-brain patients), provide a unique opportunity to investigate the function and role of CC in FC. Previous observations made in patients with intractable epilepsy who have undergone the therapeutic section of the CC report apparently conflicting results (Johnston et al., 2008; Uddin et al., 2008; Pizoli et al., 2011; Roland et al., 2017), which could be resolved by considering the age at which patients underwent surgery. Patients undergoing the callosotomy in adulthood could more likely maintain FC, thanks to the greater maturity of the brain in finding alternative pathways among the existing ones; however, not all studies in the literature confirm this hypothesis, which needs to be investigated through further studies (see Discussion section).

The present study aims to contribute to the understanding of interhemispheric FC by analyzing fMRI resting-state data of callosotomized patients, most of whom operated in adult age. Preliminary results of this research have been presented previously (Marcantoni et al., 2023).

## Materials and methods

### Participants: callosotomized patients

**Figure 1:**
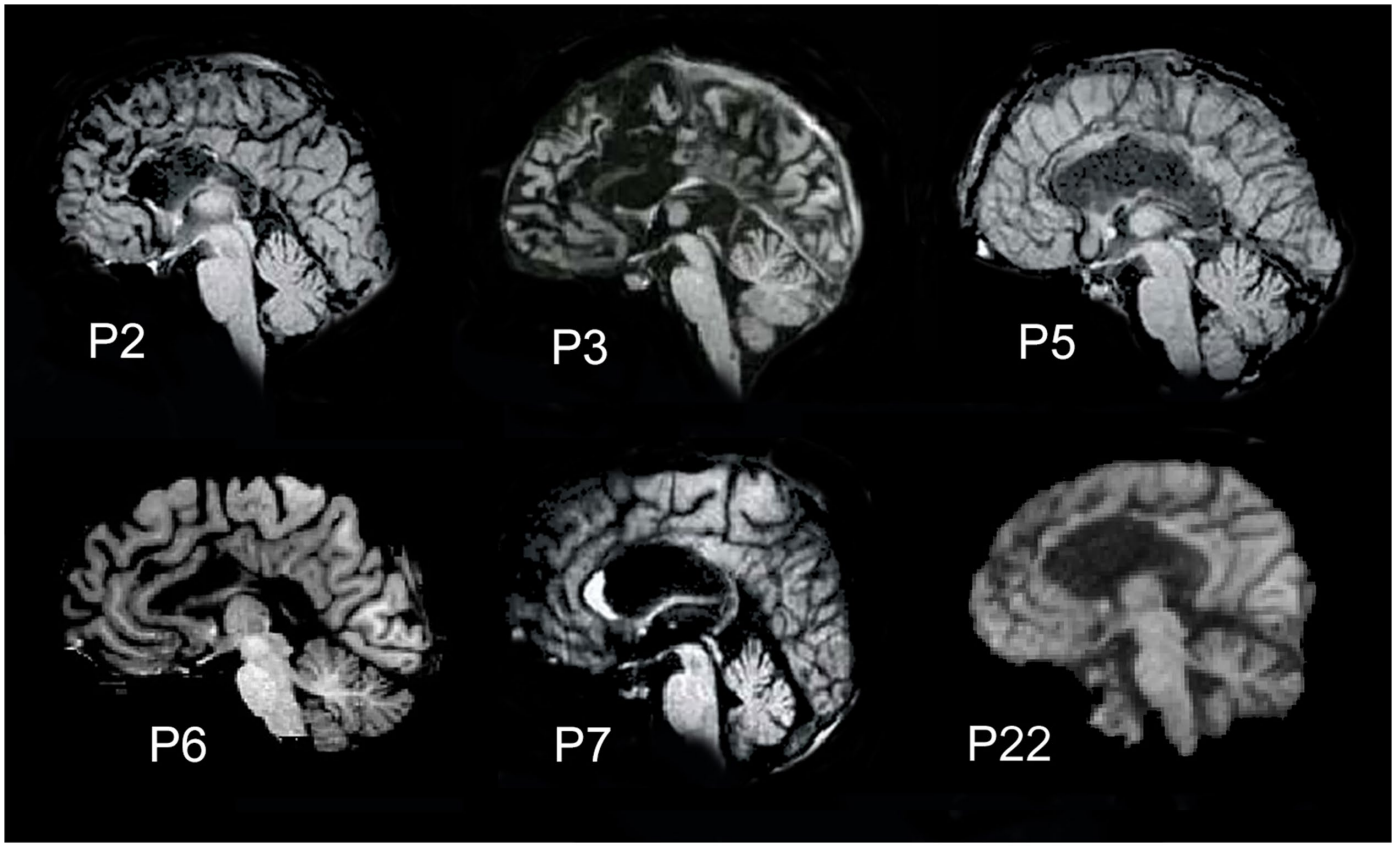
Midsagittal magnetic resonance images showing the extent of callosal resection. Five patients (P2, P3, P5, P6, and P22) carried total callosal resection; patient P7 had a partial posterior resection.

The fMRI data from six epileptic patients (40–60 years old; 2 female and 4 male patients; all right-handed) were considered. They underwent callosal resection performed in two stages, the second of which was carried out 18–24 years before the study to treat drug-resistant epilepsy. Five of the six callosotomized patients underwent total callosal resection (P2, P3, P5, P6, and P22); the sixth displayed a partial posterior callosotomy (P7). No other commissures were affected in these patients, except for patient P3, in whom part of the AC was also resected. Sagittal MR anatomical images of the patients’ brains are shown in Figure 1. Patients’ fMRI data were acquired between 2018 and 2019.

The functional MRI data from four healthy subjects (S1, S2, S3, and S4; Supplementary material) and a non-callosotomized epileptic patient (P24) were also considered. The non-callosotomized epileptic patient was initially considered for callosotomy, but later, it was not performed since it was considered unsuitable. S1, S2, S3, and S4 were considered with the main purpose to validate the analysis procedure followed and not to compare the results obtained by their data with those of split-brain patients. P24 was considered to assess whether drug therapy can only affect connectivity. However, since the authors recognize that reporting results from a few control subjects and a patient without callosotomy could be confusing to the reader, they have been omitted from the main text and provided as Supplementary material.

Details of callosotomized patients are reported in Table 1, while those of healthy subjects (controls) and non-callosotomized patient are reported in Supplementary Table S1. All participants gave their informed consent to the experimental procedure, approved by the ethical committee of Università Politecnica delle Marche.

**Table 1:** Data of callosotomized patients participating in the study.

Participants^ξ^	Gender	Age at testing	Callosal resection	Age at first surgery	Age at second surgery	Years from last surgery	Oldfield score^†^
*P2*	M	53	Total	22	29	24	10
*P3*	M	42	Total	18	19	24	21
*P5*	F	40	Total	16	17	23	10
*P6*	F	46	Total	24	26	20	10
*P7*	M	60	Partial posterior	40	41	18	10
*P22*	M	46	Total	14	22	24	10

^ξ^Participants are indicated with an alphanumeric identifier, where “P” stands for “patient” and numbers follow the order of fMRI data acquisition [for the patients, this is in accordance with the article by Fabri and Polonara (2023)]; P22 is not present in the table referenced, in that, the patient did not participate in the studies described in the article by Fabri and Polonara (2023).^†^The scores can vary between 10, which means pure right-handed, and 50, which means pure left-handed.

### Data acquisition

The patients were instructed in advance to lie down and remain still as much as possible during the MRI acquisition; they were invited to keep their eyes open, to relax without falling asleep, and without focusing on anything. Resting-state BOLD fMRI data and T1-weighted structural images were acquired using a 1.5 Signal HDxt GE Medical System MRI scanner.

The functional images were acquired with an echo planar image (EPI) gradient-echo sequence, with the following parameters: echo time (TE) = 50 ms, repetition time (TR) = 3,000 ms, flip angle = 90°, field of view (FOV) of 192 × 192 mm, matrix size of 64 × 64, number of volumes = 300, number of axial slices = 35, slice thickness = 4 mm, with no gap between slice acquisition, and voxel resolution 3 × 3 × 4 mm. The duration of the resting-state fMRI run was 15 min and 10 s. The T1-weighted structural images (3D; high-resolution whole-brain images, also called structural images) were acquired with an MPRAGE sequence with TE = 6.7 ms, TR = 14.7 ms, flip angle = 15°, FOV of 290 × 290 mm, matrix size of 512 × 512 mm, number of sagittal slices = 158, slice thickness = 1 mm with no gap between slice acquisition, and voxel resolution 1 × 0.5664 × 0.5664 mm. The duration was 8 min and 8 s.

Details about the acquisition parameters of functional and structural MRI images of control subjects and non-callosotomized patient are reported in the Supplementary material.

### Data analyses

#### Resting-state networks

Anatomical and functional scans were processed using the FMRI of the Brain (FMRIB) Software Library (FSL) for a single-subject analysis of resting-state patterns of activation (RSNs) (Smith et al., 2004; Woolrich et al., 2009; Jenkinson et al., 2012).

A preliminary preprocessing phase was necessary on anatomical and functional scans to remove unwanted artifacts and make them suitable for the following analysis. The preprocessing of functional data involved 5 steps: (1) removal of the first volumes; (2) motion correction; (3) slice timing correction; (4) spatial smoothing; and (5) temporal filtering. These steps were performed using the Multivariate Exploratory Linear Optimized Decomposition into Independent Components (MELODIC) tool (Beckmann and Smith, 2004). The first 5 volumes were deleted to exclude the initial 15 s of acquisition (15 s over the total 900 s) to guarantee stabilization of data. Motion correction was applied by turning on the MCFLIRT (an acronym that stands for Motion Correction by FMRIB’s Linear Image Registration Tool) option to correct for changes in head position while scanning. Through MCFLIRT, a rigid body transformation (i.e., via rotations and translations) was used to spatially re-align all volumes in a time series to match the middle volume, which was considered as a reference. Slice timing correction was performed considering the inter-slice delay introduced by the sequential nature (in ascending order) of the fMRI acquisition: the tool implemented a low-order Hanning windowed sinc interpolation to move each time series by a specific TR fraction with respect to the middle of the TR period. The level of spatial smoothing depends on the size of the Gaussian kernel applied to fMRI data, i.e., the full width at half maximum. To reduce noise without biasing neural activations and assuming that the identified resting-state active brain area would have been several millimeters, the full width at half maximum was set at 5 mm. Temporal filtering was performed by applying a high-pass filter with a cutoff frequency of 0.01 Hz to preserve the low-frequency (0.01–0.1 Hz) resting-state fluctuations of the BOLD signal. The normalization set-up in MELODIC was a “full search” to guarantee careful correspondence with anatomical structures, and 12 degrees of freedom were chosen for the allowed movement. The co-registration was performed using the boundary-based registration method. After preprocessing, the probabilistic independent component analysis (PICA) decomposition was performed within MELODIC to split the data of functional images into non-Gaussian sources (Beckmann and Smith, 2004). The PICA model adds a noise/error term to the model implemented by basic independent component analysis (ICA), guaranteeing improved performance. Moreover, it provides the statistical significance (Z statistics) of the assessed brain-activity spatial maps for each voxel. PICA decomposition was applied with the Z statistics threshold defining active voxel set at 0.5, while the number of independent components (ICs) was automatically assessed by the tool. The ICs in output from MELODIC were still in the native subject space since the MELODIC registration settings at the single-subject level simply generate (without applying them) transformations/warps necessary to adjust the functional data (specifically ICs) to the anatomical one, and in turn to the template atlas. The procedure of registration was effectively performed using the FMRIB’s Linear Image Registration Tool (FLIRT) (Jenkinson et al., 2002). For registration, the segmented brain was used as the subject anatomical reference. Brain extraction was previously performed using the brain extraction tool (BET) to strip the brain from non-brain structures (i.e., skull and meninges) (Smith, 2002). In the tool, the fractional intensity threshold parameter was initially set at 0.2 and adjusted for each subject where necessary. For normalization, the Montreal Neurological Institute (MNI) template atlas was used as a reference.

ICs were manually classified into noise-derived ICs and neural signal-derived ICs by visual inspection. Practically, each IC was displayed overlayed onto the normalized skull-stripped anatomical images of the subjects, and then they were labeled as “noise” or “signal,” exploiting the difference between the two classes in terms of spatial patterns. In addition, temporal and spectral characteristics were checked as further elements to consider for a reliable classification (Griffanti et al., 2017). Subsequently, the spatial patterns of ICs labeled as “signal” were specifically evaluated to recognize each of them as one of the RSNs identified by Smith (including the possibility of RSNs merging or splitting on multiple components) (Smith et al., 2009).

The major clusters of the identified RSNs were characterized in terms of extent (the number of voxels included in the activation cluster) and maximum intensity (the value of the maximum intensity within the activation cluster, expressed as Z statistics). They were obtained using the dedicated tool “cluster” of FSL.

#### Resting-state functional connectivity

Anatomical and functional scans were processed using CONN, a MATLAB/SPM-based functional connectivity toolbox (Whitfield-Gabrieli and Nieto-Castanon, 2012). The preprocessing pipeline was the standard one proposed by the toolbox (Nieto-Castanon, 2020a), flexible in possible settings, and in the present study, it was defined as analogous to the ones followed in FSL. The preprocessing of functional data involved 5 steps: (1) co-registration with correction of susceptibility distortion interactions; (2) slice timing correction; (3) outlier detection; (4) segmentation and normalization; and (5) spatial smoothing. Functional scans were first co-registered with the first scan, applying a least square approach with 6 degrees of movement freedom. Then, they were resampled by a b-spline interpolation to correct motion and magnetic susceptibility distortions. Slice timing misalignment was corrected by a sinc temporal interpolation to move each time series to a common reference time in the middle of each acquisition time. Outlier scans were detected using the Artifact Detection Tools (ART) (Whitfield-Gabrieli et al., 2011). Specifically, scans with framewise displacement (reflecting the subject/patient motion in the scanner) greater than 0.9 mm or scans that underwent changes in the BOLD time series overcoming 5 standard deviations of the global signal change, which is the absolute difference in the global BOLD signal (average over all voxels within the brain) considering two consecutive scans (Power et al., 2014). Anatomical and functional data were segmented into gray matter, WM, and cerebrospinal fluid, resampled to 2 mm isotropic voxels and the MNI-based system normalized using the tissue probability map template generated from the IXI dataset. Eventually, functional data were smoothed, setting the full width at half maximum at 5 mm. Furthermore, functional data were denoised by regression of potential confounding factors, for example, represented by WM, cerebrospinal fluid, estimated subject/patient motion, or outlier scans, and BOLD time series were bandpass filtered between 0.008 Hz and 0.09 Hz (Nieto-Castanon, 2020b).

**Figure 2:**
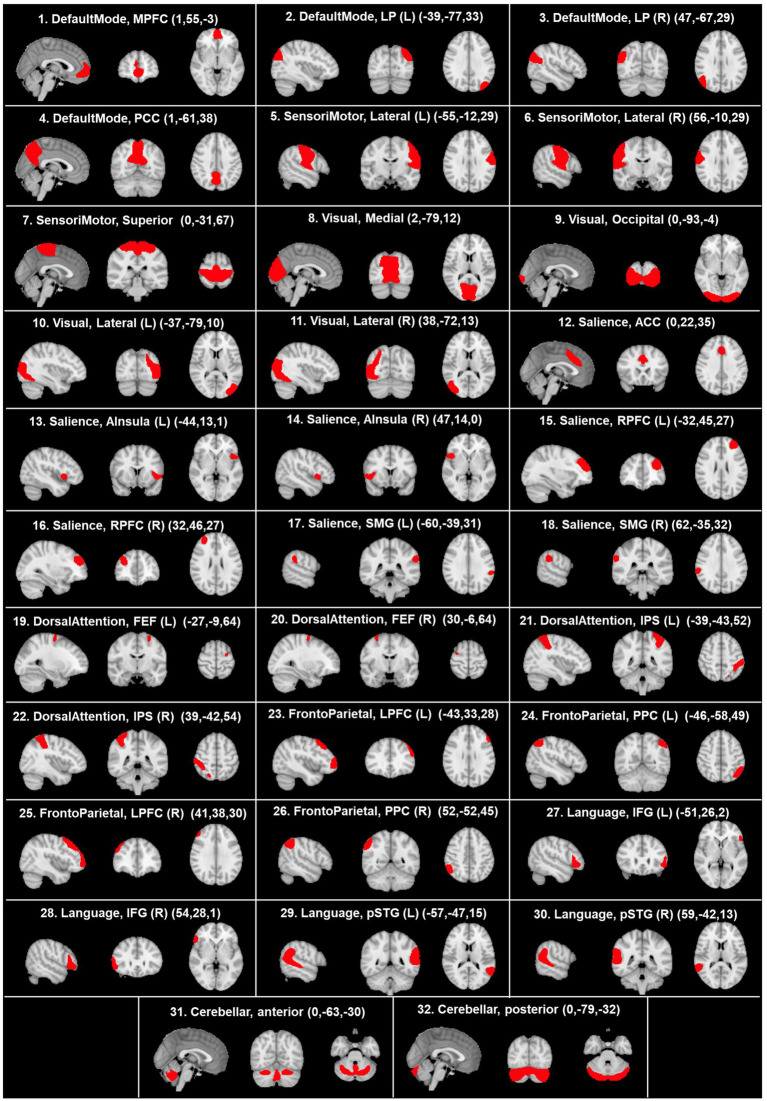
Visualization, on sagittal, coronal, and axial planes, of the morphology and positions of predefined network ROIs derived from the HCP atlas. The coordinates of each anatomical plane are indicated in parenthesis in each panel. ACC, anterior cingulate cortex; AInsula, anterior insula; FEF, frontal eye field; IFG, inferior frontal gyrus; IPS, intraparietal sulcus; LP, lateral parietal; LPFC, lateral prefrontal cortex; L, left; MPFC, medial prefrontal cortex; PCC, posterior cingulate cortex; R, right; RPFC, rostral prefrontal cortex; SMG, supramarginal gyrus; pSTG, posterior superior temporal gyrus. According to the radiological convention, the left hemisphere is on the right.

After the preprocessing and denoising phases, fMRI data underwent a regional parcellation, in which the brain was divided into 32 network regions of interest (ROIs), with predefined morphology and positions derived from the Human Connectome Project (HCP)-ICA networks (Nieto-Castanon and Whitfield-Gabrieli, 2022). The considered network ROIs are shown in Figure 2 and used for the following analysis, which evaluates the correlation of each ROI to all the others. Specifically, the mean BOLD time series was extracted within the voxels of each ROI to estimate ROI-to-ROI connectivity (RRC) matrices. Indeed, RRC matrix estimation was based on the FC strength between pairs of ROIs, quantified as the Fisher z-transformed bivariate correlation coefficients (inverse hyperbolic tangent of Pearson correlation coefficient) between the relative mean BOLD time series, exploiting a weighted general linear model (weighted-GLM) and compensating for possible transient magnetization effects (Nieto-Castanon, 2020c). Callosotomized patients’ RRC matrices were tested for statistical difference between any possible pair inside the group, applying the Wilcoxon signed rank test; statistical significance (*p*) was set to 0.05 in all cases.

The seed-based connectivity (SBC) map estimation was performed considering the right thalamus and the left thalamus as seed areas of interest, where the position and the morphology of these two areas were defined according to the Harvard-Oxford atlas ROIs (Desikan et al., 2006). In particular, the spatial pattern of FC strength between the seed area (right/left thalamus separately) and all the target voxels of the brain was characterized. Analogously with RRC matrix estimation, FC strength was quantified as the Fisher z-transformed bivariate correlation coefficients between the relative BOLD time series, exploiting a weighted-GLM and compensating for possible transient magnetization effects.

## Results

### Resting-state networks

**Figure 3:**
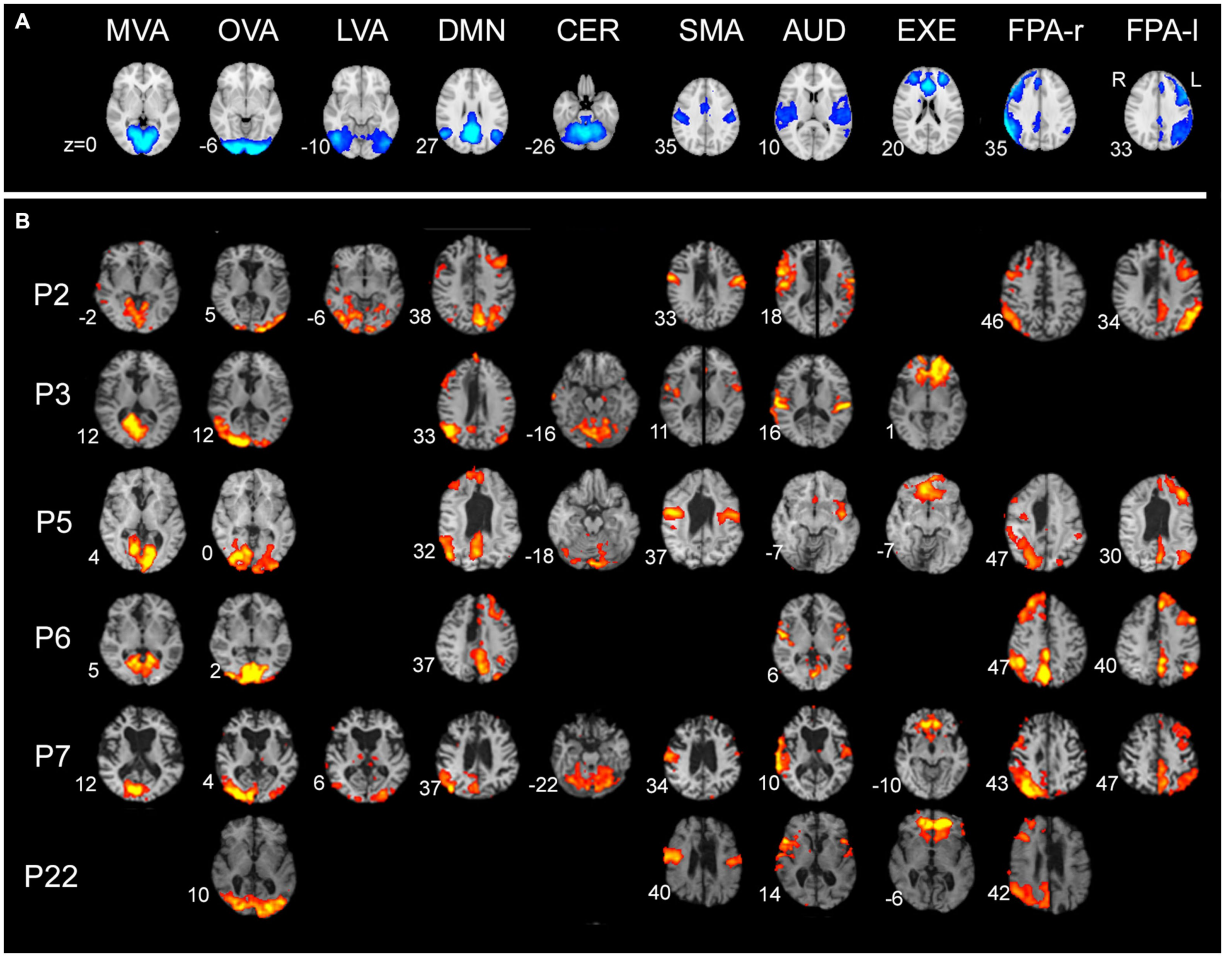
Resting-state networks in the seven patients analyzed (the lighter the color, the more intense the activation). **(A)** Networks described by Smith et al. (2009), obtained from https://www.fmrib.ox.ac.uk/datasets/brainmap+rsns/. **(B)** Resting-state networks in the seven patients analyzed showed as a result of single-subject analysis. Only patient P7 (posterior callosotomy) shows all the networks described by Smith et al. (2009). Numbers on the left of each brain figurine indicate the z-coordinate in the MNI system. According to the radiological convention, the left hemisphere is on the right.

The results obtained from callosotomized patients are reported in Table 2 and Figure 3, where the patients’ activation maps are shown. Characterization of RSNs in terms of extent (number of voxels) and maximum intensity (Z statistics) of the cluster of the activation maps are reported in Table 3.

**Table 2:** Resting-state networks reported in callosotomized patients.

	MVA	OVA	LVA	DMN	CER	SMA	AUD	EXE	FPA-r	FPA-l
P2	●	●	●	●		●	●		●	●
P3	●	●		●	●	●	●	●		
P5	●	●		●	●	●	●	●	●	●
P6	●	●		●			●		●	●
P7	●	●	●	●	●	●	●	●	●	●
P22		●				●	●	●	●	

MVA, medial visual area; OVA, occipital visual area; LVA, lateral visual area; DMN, default mode network; CER, cerebellum; EXE, executive control; SMA, sensory-motor area; AUD, auditory cortices; FPA-r, frontoparietal area right; FPA-l, frontoparietal area left.

**Table 3:** Characterization of resting-state networks in callosotomized patients: extent (voxels) and maximum intensity (Z statistics) of clusters of activation maps.

		P2	P3	P5	P6	P7	P22
MVA	*Extent*	8,245	7,988	11,178	11,322	8,335	
	*Intensity*	14.8	18.6	12.3	16.6	18	
OVA	*Extent*	5,091	13,731	14,452	14,117	9,920	16,890
	*Intensity*	12.4	20.6	21.3	41.1	18.8	12.3
LVA	*Extent*	11,859				7,537	
	*Intensity*	11.4				13.6	
DMN_1	*Extent*	5,186	4,242	6,298	14,325	4,909	
	*Intensity*	8.49	12.7	11.1	15.4	13.4	
DMN_2	*Extent*	1,937		5,613			
	*Intensity*	6.72		10.7			
CER	*Extent*		8,720	2,418		14,039	
	*Intensity*		8.57	10.5		10.3	
EXE	*Extent*		14,764	6,370		3,060	6,764
	*Intensity*		13.1	11.9		13	22.2
SMA_R	*Extent*	1,762	6,264	2,393		2,072	3,436
	*Intensity*	11	19.1	11		8.91	8.73
SMA_L	*Extent*	2,653	4,772	1,406		373	1,039
	*Intensity*	11.3	21.8	6.68		6.1	6.16
AUD_R	*Extent*	9,469	4,399	1,252	1,221	4,362	7,794
	*Intensity*	18.9	9.27	7.75	21.1	14.6	8.4
AUD_L	*Extent*	3,351	1,437	2,111	2,585	833	1,061
	*Intensity*	12.6	10.1	9.04	18.1	6.96	6.16
FPA-r_1	*Extent*	1,584		536	11,076	2,615	5,007
	*Intensity*	7.93		4.21	15.2	8.69	10.1
FPA-r_2	*Extent*	8,941		7,062	8,208	8,791	9,286
	*Intensity*	13.6		7.35	23.1	23.9	9.22
FPA-l_1	*Extent*	6,171		9,796	10,890	4,095	
	*Intensity*	12.8		14.7	17.6	6.67	
FPA-l_2	*Extent*	9,436		4,830	8,037	8,988	
	*Intensity*	14.6		9.65	12	11.9	

MVA, medial visual area; OVA, occipital visual area; LVA, lateral visual area; DMN_1, default mode network parietal area and posterior cingulate cortex; DMN_2, default mode network frontal area; CER, cerebellum; EXE, executive control; SMA_R, sensorimotor area right; SMA_L, sensorimotor area left; AUD_R, auditory area right; AUD_L, auditory area left; FPA-r_1, frontoparietal area right (frontal cortex); FPA-r_2, frontoparietal area right (parietal cortex); FPA-l_1, frontoparietal area left (frontal cortex); FPA-l_2, frontoparietal area left (parietal cortex).

Overall, all Smith’s RSNs could be recognized, although there were some differences in the presence, intensity, and bilaterality of the activations (Table 2).

All patients showed bilateral activation in primary visual areas (OVA), with a predominant activation in the right hemisphere in two patients (P3 and P7). All patients showed bilateral activation in AUD, with a predominant activation in the right hemisphere in four patients (P2, P3, P7, and P22) and a predominant activation in the left hemisphere in one patient (P5). Five patients (P2, P3, P5, P7, and P22) showed bilateral activation in SMA, with a predominant activation in the right hemisphere in three patients (P3, P7, and P22). Five patients (P2, P3, P5, P6, and P7) also showed bilateral activation in MVA, with a predominant activation in the right hemisphere in two patients (P3 and P7). DMN was observed in five patients: two of them (P2 and P3) showed bilateral activation, predominant in the left hemisphere in P2 and in the right in P3; in the remaining three patients (P5, P6, and P7), unilateral activation was observed in this network, only in the right hemisphere in P5 and P7 and only in the left in P6. Four patients showed bilateral activation in EXE, with a predominant activation in the left hemisphere in one patient (P3). FPA-r and FPA-l were observed in five (P2, P5, P6, P7, and P22) and four patients (P2, P5, P6, and P7), respectively (Figure 3), in a single hemisphere, as expected. The activation in CER was observed in three patients (P3, P5, and P7), in all cases bilaterally. Two patients (P2 and P7) showed bilateral activation in LVA, predominant in the left hemisphere in P7.

### Resting-state functional connectivity

**Figure 4:**
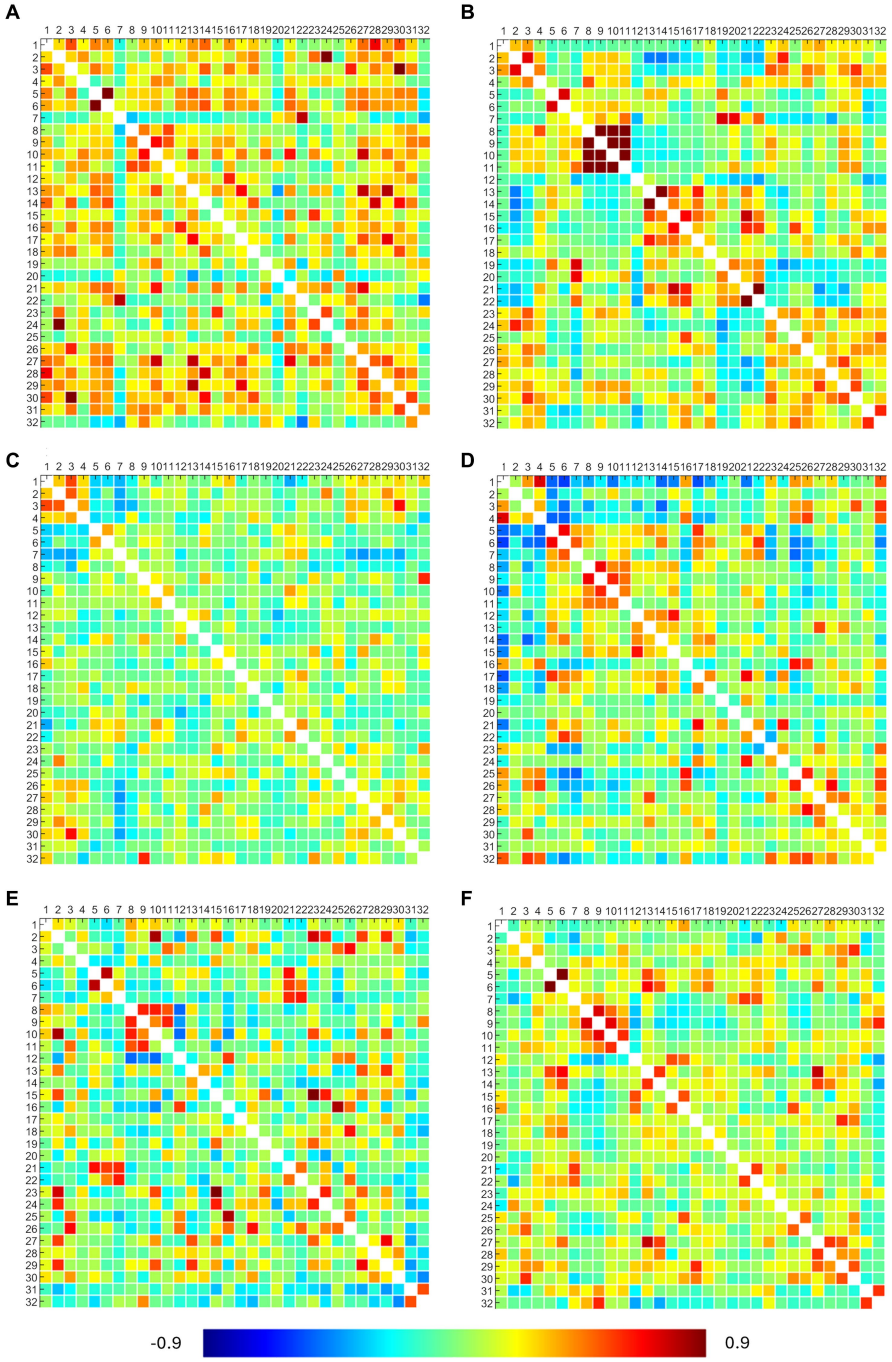
ROI-to-ROI connectivity (RRC) matrices of patients. Each panel refers to a patient, specifically: panel **(A)** presents the P2 RRC matrix; panel **(B)** presents the P3 RRC matrix; panel **(C)** presents the P5 RRC matrix; panel **(D)** presents the P6 RRC matrix; panel **(E)** presents the P7 RRC matrix; and panel **(F)** presents the P22 RRC matrix. The matrices are symmetric. Axial ticks correspond to the numbers of 32 network ROIs displayed in Figure 2. The color of each element denotes the level of functional connectivity between pairs of ROIs since it corresponds to the value of the Fisher z-transformed correlation coefficient according to the color bar shown at the bottom of the figure, where toward-blue colors represent negative correlations, while toward-red colors represent positive correlations, with a range between −0.9 and 0.9.

The obtained RRC matrices from callosotomized patients are shown in Figure 4. Despite the application of the Fisher transformation, the consequent normalization effect may sometimes not be so evident because of the quite moderate values observed.

DMN showed connections between the right and left lateral parietal cortex (LPC) in P3 and between the medial prefrontal (MPFC) and posterior parietal (PPC) cortex in P6. SMA showed bilateral connections between the left and right regions in all patients (but not with the superior region), confirming what was already observed from activation maps. Visual networks showed high connections, but right–left LVA connections were found only in P3, P6, and P22. EXE showed bilateral connections in anterior insula regions and rostral prefrontal cortex (RPFC) in P3 and P22 (lateral connections between anterior insula regions in P6 and P7). Overall, DMN and EXE showed ipsilateral connections with other cortical regions, tendentially with frontoparietal and language ones. FPA-r showed connections with right RPFC in P3, P6, P7, and P22; DMN right LPC in P2, P7, and P22; and language regions and posterior cerebellum in P6. FPA-l showed connections with DMN left LPC in P2, P3, and P7 and with left RPFC in P2 and P7. Language regions showed tendentially ipsilateral connections with the left anterior insula in P6, P7, and P22 and with both anterior insula (right and left) in P2. Moreover, the right language regions showed connections with DMN right LPC in P2, P3, P6, and P22.

Results from statistical analysis through the Wilcoxon signed rank test showed that in the patients’ group, RRC matrices resulted in statistical differences in more than 80% of considered pairs.

**Figure 5:**
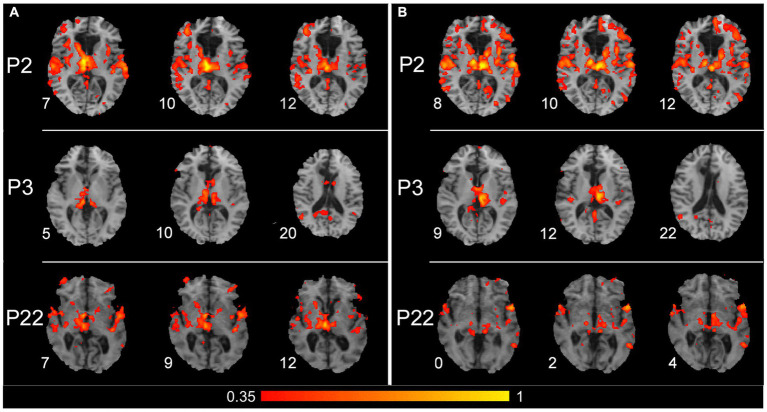
Results of the seed-based connectivity analysis, which separately considered a seed placed in the right or left thalamus. **(A)** Results obtained from P2, P3, and P22 (first, second, and third row, respectively), with a seed placed in the right thalamus. **(B)** Results obtained from the same patients, with a seed placed in the left thalamus. For all three patients, the connections between the thalamus and cortical areas are shown from three significative levels, where thalamic and/or cortical connected zones are evident. The color within the maps denotes the level of functional connectivity strength between the seed area [right and left thalamus in **(A,B)**, respectively] and the target voxels on the brain; it corresponds to the value of the Fisher z-transformed correlation coefficient according to the color bar shown on the bottom of the figure, with a range between 0.35 and 1. Numbers on the left of each brain figurine indicate the z-coordinate in the MNI system. According to the radiological convention, the left hemisphere is on the right.

The SBC analysis, which considered a seed placed in the right and left thalamus separately, seems to evidence the thalamic contribution to connectivity with contralateral cortical areas. Figure 5 shows the results obtained from three patients (P2, P3, and P22) in whom the connections from each thalamic side and contralateral cortical areas were particularly evident. The seed placed either in the right (left panels) or left (right panels) thalamus evidenced a connection with auditory cortical areas in both hemispheres in all three patients and with PPC and posterior cingulate cortex (PCC) of the right hemisphere in P3.

## Discussion

The present study investigated interhemispheric FC in the brains of patients who underwent callosal resection to alleviate severe intractable epilepsy. As already pointed out, these patients offer a unique opportunity to examine the mechanisms of interhemispheric interactions (Uddin et al., 2008). The results are based on the analysis of resting-state fMRI data to obtain RSNs and FC quantification through RRC matrices and SBC analysis. The study also involved a small population of healthy volunteers to verify that the present analysis procedure gives results comparable with those reported in the literature, and also a non-callosotomized epileptic patient to assess whether drug therapy alone can affect FC.

Present results showed that the callosotomized patients’ networks were slightly altered after callosotomy. Indeed, analysis evidenced a generally lower degree of bilateral activation, especially in some RSNs, probably due to callosotomy and/or pharmacological treatment. However, despite the callosal resection, some networks preserve a certain degree of connection between the hemispheres. Bilateral activation was observed in all patients in primary sensory cortices, i.e., in primary visual (OVA) and auditory (AUD) areas. Other sensory networks (MVA and SMA) also showed bilateral activation in almost all patients. Bilateral activation was observed in most patients in the EXE network. Nevertheless, in most cases, callosotomized patients are considered to show a predominant activation in one of the two hemispheres. In DMN only, three patients showed unilateral activation.

Results about the quantification of FC confirmed the observations derived from RSNs analysis. An evident alteration in the overall FC strength was observed among the patients. However, a consistent outcome was identified regarding the SMA, with all patients exhibiting robust FC between the lateral sensorimotor regions. Results from SBC analysis indicated a functional connection between the thalamus and the contralateral cortical region, suggesting that the thalamo-cortical could be one of the pathways contributing to the interhemispheric FC.

These results are generally consistent with previous behavioral and functional data available for some patients. Behavioral studies showed that patient P2 was able to perform interhemispheric visual transfer, evidenced by non-verbal response (Corballis et al., 2005; Pinto et al., 2017) and with specific visual stimuli wavelength (Savazzi and Marzi, 2004; Savazzi et al., 2007); P2 also evidenced a preserved ability of sound localization (Hausmann et al., 2005), indicating interhemispheric transfer of auditory stimuli. Patient P3 was able to compare tactile stimuli applied to both hands (Fabri et al., 2005) and detect and localize tactile stimuli applied by an operator anywhere on the body (de Haan et al., 2020); in addition, P3 can integrate, in a subconscious way, visual information across the two hemispheres (Pinto et al., 2017, 2023), demonstrating interhemispheric tactile and visual transfer (see also, Corballis et al., 2018).

Previous functional studies showed that bilateral activation was present in patients P2, P3, and P5, in primary and secondary somatosensory areas (Fabri et al., 2006) and the primary gustatory area (Polonara et al., 2023), despite the lack of callosal fibers connecting those areas. Bilateral activation was likely supported by subcortical bundles, clearly visible in these patients by tractography on diffusion MRI images (Polonara et al., 2023).

The difference observed across patients in preserved networks could be due to the previous personal history of each patient, the life habits, the difference in pathology severity, and the epilepsy onset age.

Few studies are available on the FC in split-brain patients, most of which are carried out on pediatric patients. Generally, these studies showed preserved FC in sensory areas; when the resection was not complete, a residual FC was also observed in cortical regions connected by the spared callosal fibers. By studying spontaneous fMRI BOLD activity in a 6-year-old child with intractable epilepsy, a striking loss of interhemispheric FC after complete callosotomy was found in all systems except in somatomotor and memory (hippocampal formation) systems (Johnston et al., 2008). Surviving somatomotor correlations may be due to ascending information transmitted via thalamo-cortical projections. Residual interhemispheric coherence in the amygdalae and anterior lateral temporal lobes in both the anterior hippocampus and hippocampal body correlation maps could be supported by the anterior and spared hippocampal commissures, where the more rostral interhemispheric and interhippocampal pathways run. In another study, analyzing spontaneous fMRI BOLD activity in a 5-year-old boy with an epileptic encephalopathy, carrying callosotomy involving the anterior 2/3 of the commissure, preserved interhemispheric FC between somatomotor cortices, in DMN, and between visual and auditory cortical areas was found (Pizoli et al., 2011). All these areas are connected by fibers likely spared from the callosotomy. At variance, the left inferior frontal gyrus (speech seed) and the left intraparietal sulcus (dorsal attention seed) lost their connections with the homotopic regions in the contralateral hemisphere. Further research analyzed the interhemispheric FC in 22 pediatric patients (2–17 years old) before and after a total (16 subjects) or partial (6 subjects) callosotomy (Roland et al., 2017). The results demonstrated that in these patients, the FC was dramatically reduced after complete callosotomy, although partially preserved homotopic FC was observed in primary sensorimotor and visual areas. Partially preserved FC, mainly in visual areas, was observed after partial callosotomy. These results suggest that structures, likely subcortical, other than the CC may contribute to FC.

The only study analyzing FC in adult patients was carried out on an old patient and reported some bilateral activation after commissurotomy, performed 4 decades before (Uddin et al., 2008). The RSNs surviving the total callosotomy were the posterior portion of DMN, comprising PCC and LPC, and the network involving the occipital cortex, referred to in the literature as OVA. The patient also showed spared visual transfer, being able to match meaningful and non-sense shapes of varying complexity and letter shapes across the vertical meridian (Eviatar and Zaidel, 1994). This is consistent with the existence of strong bilateral FC in the primary visual cortex (RSN 1, presently defined as OVA), as demonstrated here. Additionally, this patient showed preserved bilaterality in regions comprising the DMN (Raichle et al., 2001). This network is thought to be involved in various self-referential and social cognitive functions (Uddin et al., 2007). The fact that specific RSNs maintain bilateral presence after complete commissurotomy strongly suggests that their post-surgery coordination is subcortical in origin. In a recent study (Nomi et al., 2019), it was suggested that interhemispheric FC preserved in the patient described by Uddin et al. (2008) could be subserved by cortico-cerebellar connections, i.e., the cortico-ponto-cerebellar loop (CPCL) and the dentate-rubro-thalamic tract (DRTT). The CPCL consists of afferent cerebellar fibers connecting the frontal, parietal, and visual cortex to the thalamus, which then connects to the cerebellum via pontine decussations of the middle cerebellar peduncles (MCP). The DRTT consists of efferent cerebellar fibers that connect the dentate nucleus to a midbrain decussation via the superior cerebellar peduncles, which then connect to the thalamus and cortex. Evidence of substantial changes was found in fractional anisotropy of spared inter- but not intrahemispheric extra-callosal pathways in that patient, specifically in the dorsal and ventral pontine decussations. These findings demonstrate possible cortico-cerebellar mechanisms supporting preserved interhemispheric communication in commissurotomized patients based on the plasticity of existing WM tracts.

Previous studies have established that subcortical structures participate in cortical RSNs (Zhang et al., 2010; Bell and Shine, 2016). Therefore, it is likely that the most robust thalamo-cortical SC, as assessed by tractography from diffusion MRI data, which is found in primary sensorimotor and visual cortices, could underpin the interhemispheric FC observed in primary sensory areas and not in multimodal association areas, where the weakest thalamo-cortical connections are found (Toulmin et al., 2015).

In non-human primates, it has been demonstrated that cortical systems associated with the performance of sensory, motor, and/or cognitive tasks manifest spontaneous BOLD fluctuations, indicating interhemispheric connectivity between cortical areas also in the absence of normal perception or behavior (Vincent et al., 2007). In addition, residual interhemispheric connectivity was observed if AC was intact (O’Reilly et al., 2013). Furthermore, O’Reilly and coworkers found that remaining intact structural connections strengthen their FC. It was concluded that FC is likely driven by corticocortical WM connections, but in the absence of CC, complex network interactions can be maintained by just a few indirect structural pathways.

It may be inferred that homotopic FC in primary cortical areas is less dependent on the CC. This anatomy is consistent with the residual FC reported by the authors. Further evidence of indirect FC after partial callosotomy is provided. Homotopic FC in multimodal areas of the frontal lobes is reduced after callosotomy, although the reduction was less after partial callosotomy relative to complete, despite callosal fibers connecting these areas being sectioned in both procedures. This contrasts with residual interhemispheric FC in the posterior parietal and occipital cortex, which is expected from known structural connections in the splenium (Chao et al., 2009). This finding suggests that posterior parietal-occipital areas, the callosal fibers spared by partial callosotomy, can support frontal homotopic FC via intrahemispheric anatomic connections, e.g., via the superior longitudinal fasciculus. Thus, the posterior areas with maintained callosal SC act as hubs between widely separated regions in the posterior and anterior parts of the brain. These findings help to explain the absence of disconnection syndrome after partial callosotomy, where interhemispheric information transfer remains when the splenium is spared (Gordon et al., 1971).

All these results from previous and present studies share the observation of the presence of interhemispheric FC in primary sensory areas, as well as after complete callosotomy. This finding can be explained by the contribution of the thalamo-cortical input, which is generally stronger in the primary than in multimodal association areas, as suggested by the SBC analysis on the thalamus (see Results section).

In another study on an adult patient (Uddin et al., 2008) and the present, FC was also found to persist between multimodal association cortical areas in different hemispheres usually connected by the CC, such as the EXE network and DMN. This finding could be explained by considering that the brain reaches its maturity around 20 years, and therefore, it can be supposed that at this time, other communication routes between the two hemispheres, possibly alternative to the CC, are fully developed. Thus, it is possible that if callosotomy occurs around 20 years or later, these neural pathways, which are completely developed, can support the residual interhemispheric FC also observed in patients with total callosotomy. On the contrary, when the callosotomy is performed before 20 years when the brain development is not completed yet, the alternative routes, other than CC, do not have the time to be completely developed, or the brain does not have the time to learn how to use them.

This hypothesis could be evaluated in future studies, hopefully involving larger populations, by analyzing RSNs in adult patients who underwent complete callosotomy in childhood to verify whether they still lack FC. In addition, future investigations could expand the thalamic connections and evaluate the cerebellar connections with contralateral cortical regions to confirm the cerebellum involvement as alternative pathways, especially exploited in the absence of CC, as recently suggested (Nomi et al., 2019). The results obtained here can be completed by confirming them with the study of SC through the diffusion-weighted MRI data (Gollo et al., 2018) and by the depth of the FC here considered, resulting from the bivariate correlation matrix, through the effective connectivity, resulting from partial correlation matrix (Salvador et al., 2005), which keeps into account the causal contribution that the cortical regions exert over another.

## Conclusion

The present results suggest that in healthy subjects’ brains, a possible dual mechanism does exist, according to which cortical and sub-cortical pathways work together to coordinate networks. The persistence of some degree of interhemispheric FC after callosotomy shows that the CC is not the only pathway supporting this function. It can be hypothesized that the residual FC is underpinned by thalamo-cortical pathways and/or cerebello-cortical pathways via pontine/thalamic nuclei.

### Limitations of the study

Some limitations of the study should be highlighted to be overcome in future studies where possible:

- The intensity of the magnetic field is lower than in studies in the literature (1.5 T vs 3 T);
- The components were classified manually: this was necessary as the patients considered were unique; therefore, in this case, the manual classification by an expert becomes the gold standard;
- Noise and artifacts often remain despite the preprocessing and overlap the data of interest, affecting the identification of RSNs;
- fMRI data have low resolution, and this made the registration phase more complex;
- The control and patient populations are small: callosotomized patients are rare, and, on the other hand, even if small, the control population allowed the proposed procedure to be validated since the results obtained coincided with those from the literature.

## Data availability statement

The original contributions presented in the study are included in the article/Supplementary material, further inquiries can be directed to the corresponding author.

## Ethics statement

The studies involving humans were approved by Comitato Etico Regionale delle Marche. The studies were conducted in accordance with the local legislation and institutional requirements. The participants provided their written informed consent to participate in this study.

## Author contributions

IM: Conceptualization, Investigation, Supervision, Writing – original draft, Writing – review & editing, Formal analysis, Methodology, Software. GPi: Formal analysis, Investigation, Methodology, Software, Writing – review & editing. MG: Investigation, Methodology, Resources, Writing – review & editing. MV: Methodology, Data curation, Formal analysis, Software, Writing – review & editing. LR: Methodology, Software, Writing – review & editing. FM: Validation, Writing – review & editing, Formal analysis, Software, Supervision. NF: Resources, Validation, Writing – review & editing, Data curation. SL: Supervision, Validation, Writing – review & editing, Resources. LB: Supervision, Validation, Writing – review & editing, Methodology, Software. MF: Conceptualization, Investigation, Supervision, Validation, Writing – review & editing, Writing – original draft. GPo: Conceptualization, Data curation, Investigation, Methodology, Project administration, Resources, Supervision, Validation, Writing – review & editing.
