# Supplementary material for: Interhemispheric functional connectivity: an fMRI study in callosotomized patients
Source: Front Hum Neurosci. 2024 May 15;18:1363098. doi: 10.3389/fnhum.2024.1363098 (PMC11133720; doi:10.3389/fnhum.2024.1363098)
Supplement: Supplementary file 1 [file Data_Sheet_1.pdf]

SUPPLEMENTARY MATERIALS

Participants: control subjects and non-callosotomized epileptic patient

The fMRI data from four healthy subjects' (31-64 years; 3 females and 1 male; all right-handed) and one non-callosotomized epileptic patient (P24) were considered. Control subjects' fMRI data were acquired in 2022. Details of control subjects and non-callosotomized patient are reported in Table S1.

TABLE S1. Data of healthy subjects and non-callosotomized patient participating in the study

Participants[ξ]	*Gender*	*Age at Testing (years)*	*Oldfield Score†*
Controls			
S1	F	31	16
S2	M	56	12
S3	F	52	10
S4	F	64	10
Non-callosotomized patient			
P24	F	30	12

[ξ*]Participants are indicated with an alphanumeric identifier, where "S" stands for "subject" and "P" stands for "patient" and numbers follow the order of fMRI data acquisition (for the patient this is in accordance with the article by Fabri and Polonara (2023), even if P24 is not present in the table referenced, in that the patient did not participated in the studies described in the article by Fabri and Polonara (2023).
S, healthy subject; P, epileptic patient; na: not applied.
†The scores can vary between 10, which means pure right-handed, to 50, which means pure left-handed.

Data acquisition

The acquisitions were performed in different periods for patients and control subjects considered in the present study. Consequently, changes in the MRI machine software led to a change in slice thickness between patients and control subjects. The non-callosotomized patient's functional and structural images were acquired in the same way as those of callosotomized patients (see main text). The control subjects' functional images were acquired with an echo planar image (EPI) gradient-echo sequence, with the following parameters: echo time (TE) = 50 ms, repetition time (TR) = 3000 ms, flip angle = 90°, field of view (FOV) of 240×240 mm, matrix size of 64×64, number of volumes = 300, number of axial slices = 35, slice thickness = 5 mm, with no gap between slice acquisition, voxel resolution 3.75×3.75×5 mm. The control subjects' T1-weighted structural images (3D; high-resolution whole-brain images, also called structural image) were acquired with a MPRAGE sequence with TE = 6.7 ms, TR = 14.7 ms, flip angle = 15°, FOV of 290×290 mm, matrix size of 512×512 mm, number of sagittal slices=158, slice thickness=1 mm with no gap between slice acquisition, voxel resolution 1×0.5664×0.5664 mm. Durations were the same for both patients and control subjects (see main text).

Data analyses

Data analyses performed on the control subjects were intended to validate the procedure followed and not to compare the results obtained from these subjects with those from the patients, while data analyses on the non-callosotomized patient was intended to assess whether drug therapy alone can affect connectivity.

Resting-state networks. The single subject analysis on control subjects was performed in the same way as patients (including non-callosotomized patient, see main text), except for the co-registration, which was performed with the 12 degree of freedom registration method for control subjects. Moreover, a further multi-subject analysis was performed only on control subjects. The multi-subject analysis was performed after manual cleaning up of the pre-processed functional data, by removing the components classified as noise in the single-subject analysis, using the dedicated function of FSL. Then, the preprocessed cleaned functional data were registered applying the transformations/warps generated by MELODIC setting it as for the single-subject analysis, with the only difference of a non-linear normalization (instead of a linear one performed for single-subject analysis). Eventually, a temporally concatenated group ICA was applied on these preprocessed, cleaned, and registered functional data. Differently from the single-subject analysis, in which the ICA dimensionality is set automatically by the tool, in multi-subject analysis the dimensionality of the group ICA was set at 50. The procedure of manual IC labelling was reiterated, to identify common spatial maps of brain activity, which can be used as term of comparison for the spatial maps obtained in the literature (Smith et al., 2009).

Resting-state functional connectivity. The analysis was performed in the same way as callosotomized patients (see main text).

Results

The results obtained from control subjects and non-callosotomized patient are reported in Figures S1 and S2. Both the single- and multi-subject analysis on control subjects allowed the identification of all networks previously described by Smith and co-workers (Smith et al., 2009), although the networks found with the multi-subject analysis resulted sometimes less clean than expected, possibly due to the small number of control subjects. Single-subject analysis on non-callosotomized patient also allowed the identification of all networks previously described by Smith and co-workers (Smith et al., 2009). Moreover, the non-callosotomized patient showed bilateral activation in all RSNs, in accordance with control subjects but differently from callosotomized patients (main text, Figure 2).

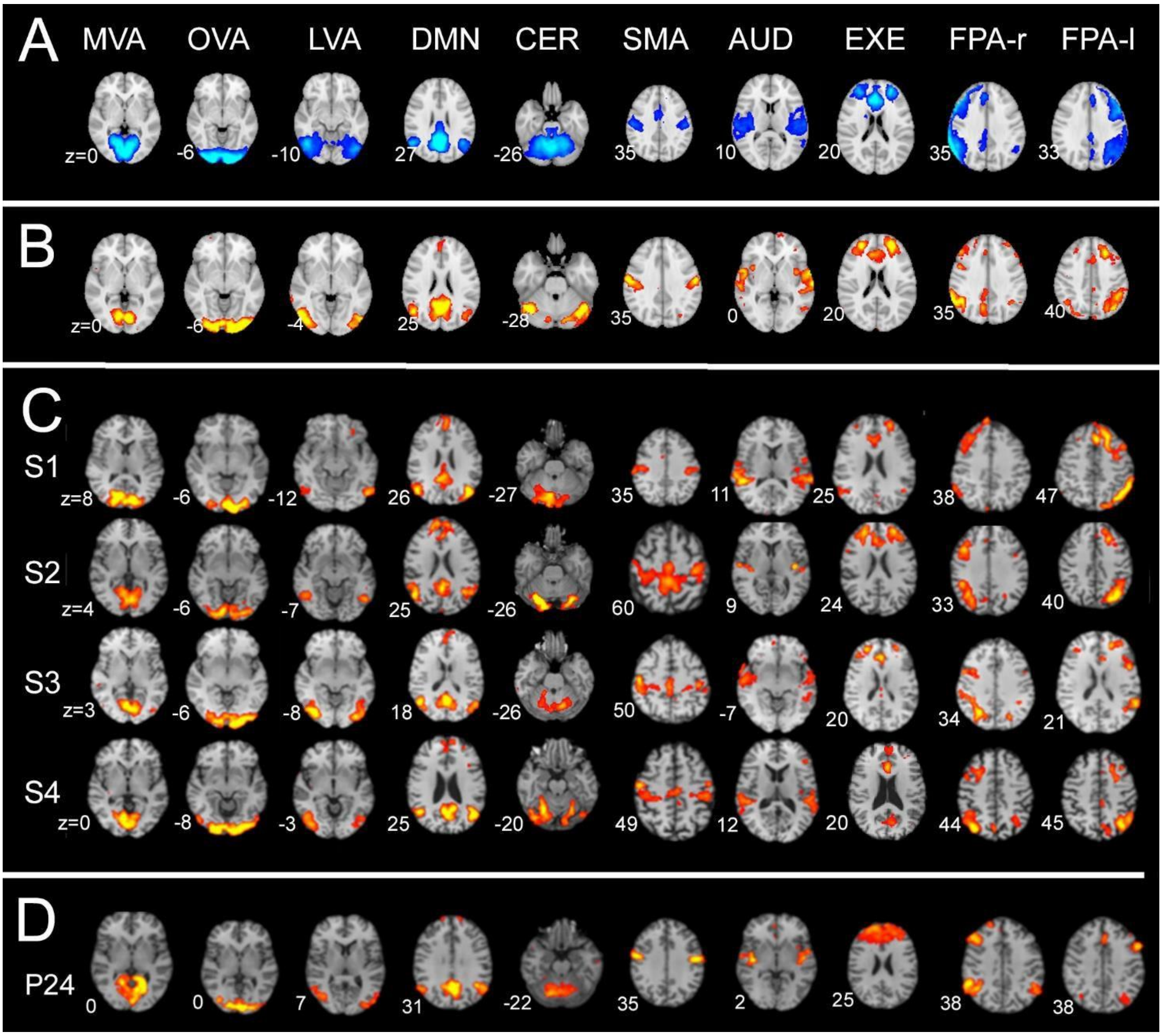

Figure S1. Resting state networks in the four control subjects and non-callosotomized patient. A, networks described by Smith and coworkers (2009), obtained from https://www.fmrib.ox.ac.uk/datasets/brainmap+rsns/. B, networks in control subjects as resulted from multi-subject analysis. C, networks in control subjects as resulted from single-subject analysis. D, networks in non-callosotomized patient as resulted from single-subject analysis. In all cases, it was possible to observe all the networks described by Smith and coworkers (2009; A). Numbers on the left of each brain figurine indicate the z coordinate in MNI system. According to the radiological convention, the left hemisphere is on the right.

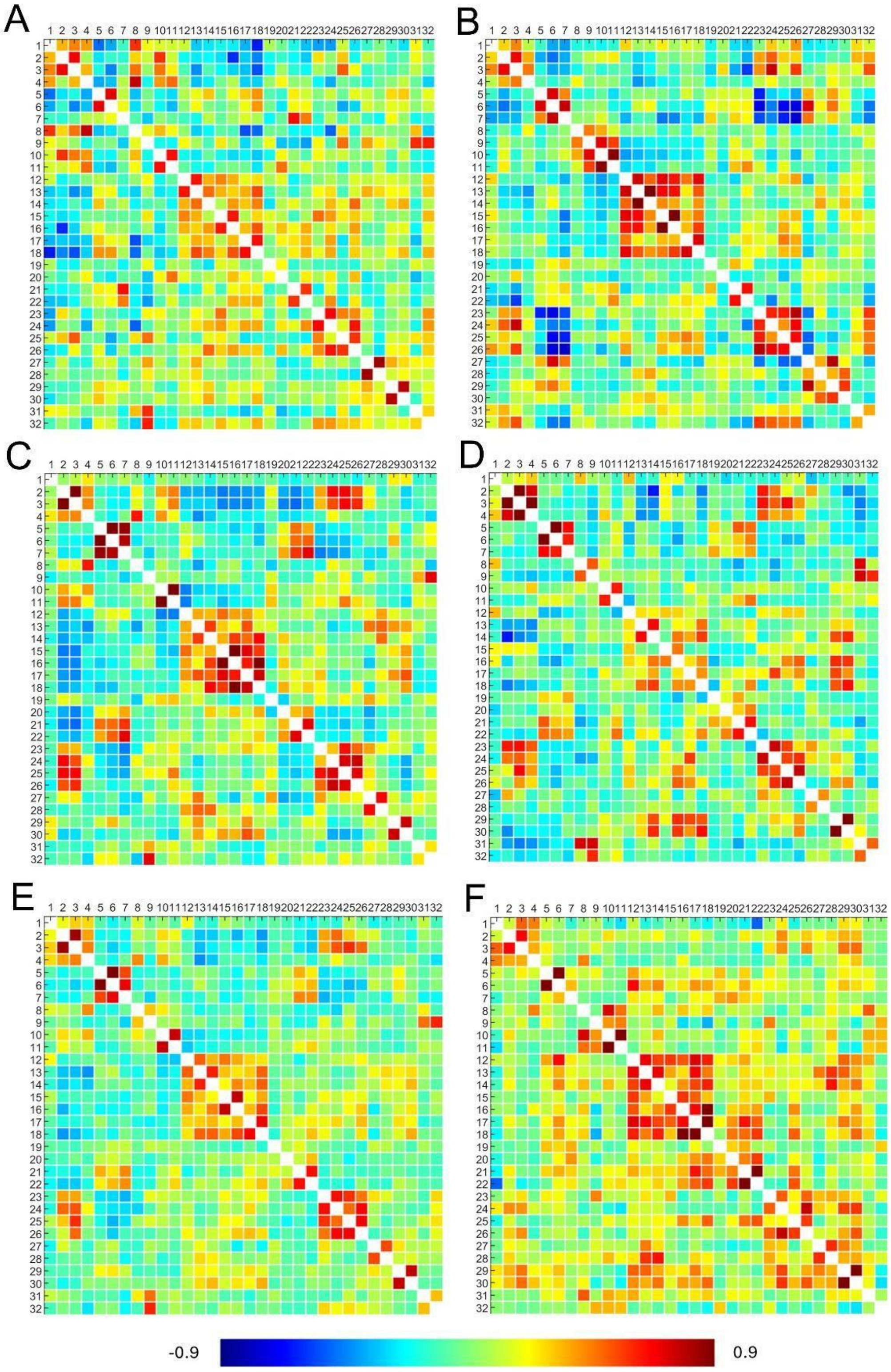

Figure S2. ROI-to-ROI connectivity (RRC) matrices of healthy control subjects and non-callosotomized patient. Panels A, B, C, D, show RRC matrices of subjects S1, S2, S3, S4, respectively. E, median of RRC matrices of the four subjects S1-S4. F, RRC matrix of patient P24. The matrices are symmetric. Axial ticks correspond to the numbers of 32 network ROIs displayed in Figure 2. The color of each element denotes the level of functional connectivity between pairs of ROIs, since it corresponds to the value of the Fisher z-transformed correlation coefficient according to the color bar shown on the bottom of the figure, where toward-blue colors represent negative correlations, while toward-red colors represent positive correlations, with a range between -0.9 and 0.9.
